# Negative Pressure Incision Management System in the Prevention of Groin Wound Infection in Vascular Surgery Patients

**DOI:** 10.1155/2015/303560

**Published:** 2015-11-01

**Authors:** Jan H. Koetje, Karsten D. Ottink, Iris Feenstra, Wilbert M. Fritschy

**Affiliations:** Department of Vascular Surgery, Isala Zwolle, 8025 AB Zwolle, Netherlands

## Abstract

*Objectives*. Groin wounds following vascular surgery are highly susceptible to healing disturbances, with reported site infections reaching 30%. Negative pressure incision management systems (NPIMS) are believed to positively influence the prevention of surgical wound-healing disturbances (WHD) and surgical site infections (SSI). NPIMS placed directly after closure of the surgical wound is thought to result in fewer infections; we analysed its effect on postoperative wound infections in patients after vascular surgery via the groin. *Methods*. From May 2012 to March 2013 we included 90 surgical patients; 40 received a NPIMS. All patients with WHDs were labelled and subanalysed for surgical site infection in case of positive microbiological culture. These infections were graded according to Szilagyi. Number of WHDs and SSIs were compared across cohorts. *Results*. Patient and perioperative characteristics were equal, except for a significantly higher number of emergency procedures among non-NPIMS patients. We found no significant differences in number of WHDs, SSIs, or Szilagyi grades between the two cohorts. *Conclusion*. The equal number of SSIs across cohorts showed that NPIMS could not reduce the number of surgical site infections after vascular groin surgery.

## 1. Introduction

Negative pressure incision management systems (NPIMS), such as Prevena (KCI USA, Inc., San Antonio, TX), [1] are believed to have a positive effect in the prevention of surgical wound-healing disturbances and surgical site infection. Negative pressure treatment directly after closure of the surgical wound is thought to result in a better distribution of tensile forces on the wound edges, evacuation of subcutaneous seroma and haematoma, reduction of surgical site oedema, increased microvascular blood flow, prevention of influx and invasion of microorganisms, and protection and sealing of the wound [2].

It is known that groin wounds after vascular surgery are highly susceptible to wound-healing disturbances, with reported site infections varying up to 30% as reported in previous studies [3–6].

Several studies demonstrate the preventive effect of negative pressure wound management, for instance, after sternotomy, below knee amputations, and after surgery for blunt high energy trauma of the lower leg [7–10]. It is thought that vascular surgery in the groin region shows a high rate of wound problems due to disruption of lymphatics, proximity to the perineum, and the use of prosthetic material. Matatov et al. published recently a retrospective study on the use of NPIMS on groin wounds in vascular surgery patients [3]. They found a reduction from 30% site infections in the control group to 6% site infections in the NPIMS group (*P* = .001). Based on these promising data we started to apply NPIMS on groin wounds after vascular surgery. In this study, we will describe our first experience with this negative pressure wound management system.

## 2. Methods

Since early 2012, the Department of Vascular Surgery of our hospital has taken part in a Nationwide Hospital Quality Program for the registration of complications and surgical site infections by using a fixed protocol named “Prevention of Hospital bound Infections by Surveillance” (PREZIES) [11]. The registration of groin wound-healing disturbances (WHD) in vascular surgery patients was part of this program.

The NPIMS was introduced in May 2012. It was applied routinely to patients who underwent vascular surgery through a groin incision by two out of four vascular surgeons. No further randomisation was performed. After one year we analysed the data that were prospectively collected for PREZIES. We collected additional data from the hospital database and patient charts.

Ninety consecutive patients who underwent vascular surgery in the groin were included; 40 received the NPIMS on the surgical groin wound (NPIMS group). The remaining 50 patients did not receive NPIMS (non-NPIMS group). All patients underwent some kind of vascular operation on the common femoral artery, such as local endarterectomy or vascular bypass. They received preventive antibiotics 30 minutes prior to the start of operation, and the groin wounds were surgically closed by double-layer subcutaneous suturing and skin approximation with agraves. Patients suffering Rutherford 5 and 6 received routinely antibiotics perioperatively and at least one week after operation. In case of wound-healing disorders, a microbiological culture was taken and, if needed, antibiotic therapy was adjusted. Patients and nurses were instructed on the usage of the NPIMS. According to the instructions for use, the aim was to leave the NPIMS placed on the surgical wound for a minimum of four days. After discharge, follow-up took place during outpatient visits at the Department of Vascular Surgery.

All wounds with healing disorders (including erythema and swelling) and so clinical signs of infection were graded as “wound-healing disorder” (WHD). A WHD combined with positive microbiological culture was classified as a surgical site infection (SSI). These postoperative site infections were graded according to the Szilagyi classification system (Table 1) [12].

We have compared the number of WHDs and SSIs in the two patient cohorts (non-NPIMS group and NPIMS group). Based on the number of surgical site infections of the control group in the study of Matatov et al., we have calculated our required sample size, which seemed to be at least 40 subjects per group (95% confidence interval and power 0.8). Patient data were collected and analysed in SPSS.

For quality registration, we were obliged to collect data prospectively. We retrospectively analysed our results of the previously introduced NPIMS, without experimental base. Also, this study was performed by members of the treating medical team. For these reasons there was no need for gaining informed consent. The Institutional Review Board has approved our protocol, without further obligations.

## 3. Results

Of the 90 patients, 40 received the NPIMS on the groin (44%). Patient characteristics like age, BMI, smoking behaviour, diabetes mellitus, renal failure, end-stage renal disease, colonisation of microorganisms of preexisting wounds, preexisting colonisation with multiresistant microorganisms (in this cohort only ESBL), ASA scores, and Rutherford classifications were compared. It was found that none of these parameters were significantly different between cohorts (Table 2). Perioperative characteristics were also compared, such as the use of prosthetic material (femoropopliteal bypass or aortobifemoral bypass), hybrid vascular surgery (endovascular procedure in combination with local endarterectomy of the common femoral artery), operating time, or whether the patient was operated in an emergency situation. Only the latter was found to be significantly different between cohorts; other perioperative factors were found to be comparable (Table 3).

A logistic regression was performed to analyse which patient or perioperative factors contributed to a WHD or a SSI. The analysis showed that there were no contributing patients or perioperative factors.

A WHD was found in 14.4% (*N* = 13) of analysed cases. Eight of them had positive microbiological cultures and were classified as surgical site infections (8.9%). The other WHDs were oedema, hematoma, or seroma, without bacterial infection. Of the patients with a WHD, 6 were found in the non-NPIMS group (12%) and 7 in the NPIMS group (17.5%, *P* = .552). SSIs with a positive microbiological culture were found in 3 patients in the non-NPIMS group (6%) and in 5 patients in the NPIMS group (12.5%, *P* = .458) (Table 4).

The site infections with positive microbiological cultures were scored according to the Szilagyi scale. Both cohorts displayed mainly superficial wound problems (Szilagyi grade 1). We found no significant differences in Szilagyi grades between cohorts (*P* = 1.00).  Table 5 shows the different microbiological cultures from the groin infections in each cohort.

## 4. Discussion

Incisions in the inguinal region are known for an increased risk of surgical site infection. Wound problems after vascular groin operations especially lead to major morbidity (sepsis, limb amputation), prolonged hospital stay, increased costs, and even substantial mortality [5]. In 2007, Stewart et al. conducted a meta-analysis of 34 randomised controlled trials and concluded that, besides prophylactic antibiotics for vascular surgery, there are no surgical techniques for preventing groin wound-healing problems [13]. Because of our positive experience with the vacuum wound-closure system on open surgical wounds and some auspicious studies with this vacuum system on closed surgical wounds [3], we started using this system after the promising study of Matatov et al. [3]. The NPIMS costs around 300 euros per single system, which would easily signify savings in the long run if SSIs could be prevented and hospital stay shortened.

Although some bias might be present by the selective use of the NPIMS by two out of four vascular surgeons, we have found that patient and perioperative characteristics were comparable, as shown by the analysis of the baseline characteristics of both patient cohorts. Known risk factors for wound-healing disturbances after vascular surgery, such as smoking, elevated body mass index, diabetes mellitus, and renal failure, were also found to be similar in both cohorts; even the distribution of Rutherford classification in patients operated for chronic limb ischemia was equally divided over the cohorts.

When comparing all parameters, only the number of emergency procedures differs significantly between the two groups, with higher numbers in the non-NPIMS group. This is also seen in the logistic regression that is performed. Elective surgery is a contributing factor in patients with NPIMS. This theoretically provides the NPIMS group an advantage, as emergency surgery is a risk factor for developing postoperative wound infection [5]. When subanalysing the elective operations only, we see equal results: more wound-healing disorders and surgical site infections in the NPIMS group.

In our daily practice, we have experienced failure of the NPIMS in different ways. The vacuum of the system was often failing, probably due to infolding in groin. Also movements of the leg did loosen the drape. Although there were several attempts to secure dry skin before applying the system, there were still several failures. If possible, we renewed the drape, in order to secure the vacuum. In an extra subanalysis, we found that failure of the NPIMS (which has led to application of less than four days and so did not meet the instructions for use) did not influence the number of SSIs.

Our study shows that, in any comparison of either surgical site infections or wound-healing problems, the NPIMS did not make a significant difference. Our hypothesis that wound-healing could be improved by the immediate evacuation of wound fluids through fast closure of the surgical wound under vacuum could not be demonstrated. The outcome of our study in combination with the sparse data in the literature does not support widespread application of the NPIMS on groin wounds after vascular surgery. Improvements of the system are advocated in order to avoid failure of the vacuum due to drape loosening, but, more importantly, prospective randomised clinical trials are needed to settle the value of vacuum wound management systems on closed (vascular) surgical wounds.

## 5. Conclusion

Our study on the prevention of wound-healing disturbances and surgical site infections after vascular surgery in the groin could not demonstrate any beneficial effect of the negative pressure incision management system. Improvements of the system are needed to achieve reliable and durable application of the vacuum, and prospective randomised trials are needed before widespread implementation of this costly wound dressing.

## Figures and Tables

**Table 1:** Szilagyi classification of surgical site infections.

Class	Description
Szilagyi I	Infection only involves the dermis
Szilagyi II	Infection extends into the subcutaneous tissue and does not invade the arterial implant
Szilagyi III	Arterial implant is involved in the infection

**Table 2:** Patient characteristics.

	Non-NPIMS	NPIMS	*P* value
Number of patients	50	40	
Gender			
Male^1^	34 (68.0%)	30 (75.0%)	.493^3^
Female^1^	16 (32.0%)	10 (25.0%)	.493^3^
Age^2^	71.5 ± 11.0	68.1 ± 8.6	.070^4^
BMI^2^	25.9 ± 5.8	27.4 ± 5.2	.130^4^
Smoking^1^	18 (36.0%)	23 (57.5%)	.056^3^
Diabetes mellitus^1^	15 (30.0%)	16 (40.0%)	.375^3^
Renal disorder^1^	22 (44%)	20 (50%)	.672^3^
End-stage renal disease^1^	2 (4.0%)	4 (10%)	.400^3^
Colonisation of preexisting wounds^1^	14 (28%)	10 (25%)	.814^3^
Preexisting multiresistant colonies^2^	3 (6.0%)	1 (2.5%)	.626^3^
ASA classification^1^			
I	0	0	.728^3^
II	19 (38.0%)	14 (35.0%)	“
III	31 (62.0%)	25 (62.5%)	“
IV	0	1 (2.5%)	“
Rutherford scale^1^			
I	11 (22.0%)	7 (17.5%)	.868^3^
II	2 (4.0%)	1 (2.5%)	“
III	7 (14.0%)	10 (25.0%)	“
IV	9 (18.0%)	7 (17.5%)	“
V	17 (34.0%)	12 (30.0%)	“
VI	4 (8.0%)	3 (7.5%)	

BMI: body mass index.

ASA classification: American Society of Anaesthesiologists' classification of physical health.

Data presented as either ^1^number (percentage) or ^2^mean ± standard deviation.

^3^ *P* value using Fisher's Exact Test.

^4^ *P* value using independent samples *t*-test.

**Table 3:** Perioperative characteristics.

	Non-NPIMS (*N* = 50)	NPIMS (*N* = 40)	*P* value
Procedure^1^			
Fem-pop Bypass	19 (38%)	14 (35%)	.455^3^
Hybrid endovascular surgery	9 (18%)	4 (10%)	“
Aortobifem-bifurcation	6 (12%)	3 (7.5%)	“
Endarterectomy	11 (22%)	10 (25%)	“
Other	5 (10%)	9 (22.5%)	“
Operating time (minutes)^2^	154.3 ± 51.3	162.6 ± 63.0	.154^4^
Emergency surgery^1^	17 (34%)	5 (12.5%)	.026^3^
Prosthetic material used^1^	13 (26%)	16 (40%)	.179^3^

Data presented as either ^1^number (percentage) or ^2^mean ± standard deviation.

^3^ *P* value using Fisher's Exact Test.

^4^ *P* value using independent samples *t*-test.

**Table 4:** Incidence of postoperative infection and Szilagyi grades of infection.

	Non-NPIMS (*N* = 50)	NPIMS (*N* = 40)	*P* value
Wound-healing disorder^a,1^	6 (12.0%)	7 (17.5%)	.552^2^
Surgical site infection^b,1^	3 (6.0%)	5 (12.5%)	.458^2^
Szilagyi grade 1	2 (66.7%)	4 (80.0%)	1.000^2^
Szilagyi grade 2	0	0	“
Szilagyi grade 3	1 (33.3%)	1 (20.0%)	“

^a^Seroma/hematoma/dehiscence/erythema with or without microbiological culture.

^b^With positive microbiological culture.

Data presented as ^1^number (percentage).

^2^ *P* value using Fisher's Exact Test.

**Table 5:** Microbiological cultures of groin wound infections.

	Non-NPIMS (*N* = 4)	NPIMS (*N* = 5)
*Staphylococcus aureus*	4 (100%)	2^a^ (40%)
*Streptococcus hemolyticus*	0	1^a^ (20%)
*Escherichia coli*	0	1 (20%)
*Pseudomonas aeruginosa*	0	1 (20%)
*Enterococcus faecalis*	0	1^a^ (20%)
*Enterobacter cloacae*	0	1^a^ (20%)

^a^Found in multibacterial cultures (*Strept. hem*. and *Staph. aur*. and *Ent. face*. and *Ent. cloacae*).

## References

[1] KCI Licensing (2010). *Incision Management System, Product Monograph*.

[2] Pachowsky M., Gusinde J., Klein A. (2012). Negative pressure wound therapy to prevent seromas and treat surgical incisions after total hip arthroplasty. *International Orthopaedics*.

[3] Matatov T., Reddy K. N., Doucet L. D., Zhao C. X., Zhang W. W. (2013). Experience with a new negative pressure incision management system in prevention of groin wound infection in vascular surgery patients. *Journal of Vascular Surgery*.

[4] Slappy A. L. J., Hakaim A. G., Oldenburg W. A., Paz-Fumagalli R., McKinney J. M. (2003). Femoral incision morbidity following endovascular aortic aneurysm repair. *Vascular and Endovascular Surgery*.

[5] Bandyk D. F. (2008). Vascular surgical site infection: risk factors and preventive measures. *Seminars in Vascular Surgery*.

[6] Ploeg A. J., Lardenoye J.-W. P., Vrancken Peeters M.-P. F. M., Hamming J. F., Breslau P. J. (2009). Wound complications at the groin after peripheral arterial surgery sparing the lymphatic tissue: a double-blind randomized clinical trial. *American Journal of Surgery*.

[7] Grauhan O., Navasardyan A., Hofmann M., Müller P., Stein J., Hetzer R. (2013). Prevention of poststernotomy wound infections in obese patients by negative pressure wound therapy. *Journal of Thoracic and Cardiovascular Surgery*.

[8] Masden D., Goldstein J., Endara M., Xu K., Steinberg J., Attinger C. (2012). Negative pressure wound therapy for at-risk surgical closures in patients with multiple comorbidities: a prospective randomized controlled study. *Annals of Surgery*.

[9] Stannard J. P., Volgas D. A., Stewart R., McGwin G., Alonso J. E. (2009). Negative pressure wound therapy after severe open fractures: a prospective randomized study. *Journal of Orthopaedic Trauma*.

[10] Stannard J. P., Volgas D. A., McGwin G. (2012). Incisional negative pressure wound therapy after high-risk lower extremity fractures. *Journal of Orthopaedic Trauma*.

[11] RIVM Strategisch beleidsplan PREZIES 2011–2015. http://www.rivm.nl/dsresource?objectid=rivmp:212486&type=org&disposition=inline&ns_nc=1.

[12] Szilagyi D. E., Smith R. F., Elliott J. P., Vrandecic M. P. (1972). Infection in arterial reconstruction with synthetic grafts. *Annals of Surgery*.

[13] Stewart A. H., Eyers P. S., Earnshaw J. J. (2007). Prevention of infection in peripheral arterial reconstruction: a systematic review and meta-analysis. *Journal of Vascular Surgery*.

